# Dual-Crystallizable Silk Fibroin/Poly(L-lactic Acid) Biocomposite Films: Effect of Polymer Phases on Protein Structures in Protein-Polymer Blends

**DOI:** 10.3390/ijms22041871

**Published:** 2021-02-13

**Authors:** Fang Wang, Yingying Li, Christopher R. Gough, Qichun Liu, Xiao Hu

**Affiliations:** 1 Center of Analysis and Testing, Nanjing Normal University, Nanjing 210023, China; 171135011@stu.njnu.edu.cn (Y.L.); 161135010@stu.njnu.edu.cn (Q.L.); 2 School of Chemistry and Materials Science, Nanjing Normal University, Nanjing 210023, China; 3 Department of Physics and Astronomy, Rowan University, Glassboro, NJ 08028, USA; goughc2@students.rowan.edu; 4 Department of Chemistry and Biochemistry, Rowan University, Glassboro, NJ 08028, USA; 5 Department of Biomedical Engineering, Rowan University, Glassboro, NJ 08028, USA; 6 Department of Molecular and Cellular Biosciences, Rowan University, Glassboro, NJ 08028, USA

**Keywords:** silk, PLLA, *β*-sheet, crystallinity, stability, DSC, Raman, XRD

## Abstract

Biopolymer composites based on silk fibroin have shown widespread potential due to their brilliant applications in tissue engineering, medicine and bioelectronics. In our present work, biocomposite nanofilms with different special topologies were obtained through blending silk fibroin with crystallizable poly(L-lactic acid) (PLLA) at various mixture rates using a stirring-reflux condensation blending method. The microstructure, phase components, and miscibility of the blended films were studied through thermal analysis in combination with Fourier-transform infrared spectroscopy and Raman analysis. X-ray diffraction and scanning electron microscope were also used for advanced structural analysis. Furthermore, their conformation transition, interaction mechanism, and thermal stability were also discussed. The results showed that the hydrogen bonds and hydrophobic interactions existed between silk fibroin (SF) and PLLA polymer chains in the blended films. The secondary structures of silk fibroin and phase components of PLLA in composites vary at different ratios of silk to PLLA. The *β*-sheet content increased with the increase of the silk fibroin content, while the glass transition temperature was raised mainly due to the rigid amorphous phase presence in the blended system. This results in an increase in thermal stability in blended films compared to the pure silk fibroin films. This study provided detailed insights into the influence of synthetic polymer phases (crystalline, rigid amorphous, and mobile amorphous) on protein secondary structures through blending, which has direct applications on the design and fabrication of novel protein–synthetic polymer composites for the biomedical and green chemistry fields.

## 1. Introduction

In recent years, silk fibroin (SF) extracted from the natural silkworm cocoon has attracted attention as one of the most promising bioengineering materials due to its unique molecular structure, excellent biocompatibility, morphologic flexibility, ability to promote cell adhesion and growth, and lower inflammatory response [1,2,3,4,5,6]. SF is easily transformed into various tunable morphologies such as nanofibers, thin films, and particles by controlling the secondary structure of SF proteins. However, it is difficult for a single material to fit all the requirements of a specific biological function or a desired application because of defects in individual material properties. For SF, these defects include low mechanical strength, poor stability, poor thrombogenicity, and easy hydrolysis of regenerated SF [1,7]. Therefore, an active strategy is blending different materials to enhance the overall properties to create a polymeric composite material through a synergistic combination of the desirable properties from original components [1,2,7].

For example, SF composite membranes with polyethylene glycol showed significantly improved mechanical properties in comparison to SF membranes without polyethylene glycol [8,9]. In another example, 1:1 SF/poly(L-lactic acid) (PLLA) composite films prepared by electrospinning showed high keratinocytes attachment and proliferation; plus, the material itself showed enhanced mechanical properties [7]. A novel nonwoven sheet of SF/polyurethane blending for cardiovascular tissue engineering also exhibited good miscibility and feasible mechanical properties [8,10]. These changes in the material properties are closely related to their structure and morphology. Zhang et al. [11] found that the strain rate of the composite material raised from 5% to 18% with the addition of graphene oxide to SF, which was believed to be related to the crystalline states in the structure. Our previous study [12] illustrated that a nanocomposite structure transition to a micro-composite structure in the silk and non-crystallizable polylactic acid composites occurred when the silk content comprised at least 30% of the material, which affected their mechanical properties and cell adhesion.

Like the constant enthusiasm and wide interest with silk, polylactic acid (PLA), an important member of aliphatic polyesters, has also been widely studied as a renewable green resource, similarly to beet and corn starch proteins [12,13,14]. PLA also has excellent physical properties, such as high modulus, transparency, and heat resistance. PLA-based materials are also promising biomaterials due to their high biocompatibility and low toxicity [15,16,17], while being easily processed into useful forms. Even so, the properties of PLA-based composites can be improved further by blending PLA with other materials. For instance, Bindhu et al. [13] proved that boron nitride and PLA composite films reinforced the tensile strength by about 132% that of pure PLA films.

Most of the above-mentioned research focused their applications on electrospun nanofibers at some specific ratio of SF to PLA [3,5,7,8,10,11,17]. In this work, dual-crystallizable silk fibroin/poly(L-lactic acid) (SF/PLLA) nanocomposite films at different mixing ratios were first-time obtained by using a new physical blending method. The ratios of SF were from 0% to 100% at about 10–20% intervals. Thermodynamic parameters such as glass transition temperature, specific heat, enthalpy, and general thermal stability were studied using differential scanning calorimetry (DSC), including standard DSC and StepScan DSC (SSDSC), and a thermogravimetric (TG) analysis. Scanning electron microscope (SEM) techniques, Fourier-transform infrared spectroscopy (FTIR), Raman spectroscopy (Raman), and X-Ray diffraction (XRD) were used to observe the surface morphology and microstructure of the films. Further, the percentage of secondary structures for silk fibroin was calculated, as well as the content of amorphous and crystalline structures for PLLA in the blended polymers. The interaction mechanism between the two crystallizable components is also discussed. This study provided a comprehensive overview of the microstructure transition and the relationship between thermal stability and structure in dual-crystallizable SF/PLLA nanocomposites. Different microstructures of the blended materials are associated with different physical properties. A full understanding of the blending characteristics of two materials at different proportions is of a great significance for the practical application of the materials. Based on our blending techniques, the blended materials may be subsequently designed into various structured biomaterials with tunable properties. This enables broad applications in the biomedical sciences, tissue regeneration, and controlled drug delivery in the future. For example, we already fabricated a series of tunable and biodegradable polylactide-silk fibroin 3D scaffolds, which have good cell compatibility and can support cell adhesion and tissue regeneration [18]. Simultaneously, this study focuses on understanding interactions between natural protein and crystallizable synthetic polymer components to obtain SF-based biocomposites with various and ideal functional properties.

## 2. Results and Discussion

### 2.1. Microstructure of Blended Films

**Figure 1:**
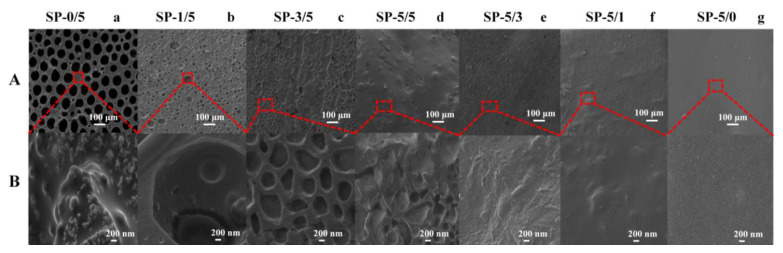
SEM images of silk fibroin/poly(L-lactic acid) (SF/PLLA) blended films within (**A**) a 100-μm scale bar and (**B**) 200-nm scale bar.

SEM was used to investigate the morphology of SF and PLLA blends with different mixing ratios (Figure 1). The morphology of all blended films was uniform on a macroscopic scale, with interesting network patterns (Figure 1A, bar scale 100 µm). For pure PLLA (SP-0/5), a relatively smooth topology with pores is observed (Figure 1Aa). Simultaneously, a dense grain structure at the junction of the pores can be observed, which may be spherulite crystals of PLLA, as seen in other studies [16]. This type of morphology can be used for tissue scaffolds to culture regenerated cells or for filter membranes to treat pollution [3,7,13,14]. Pure silk fibroin film (SP-5/0) maintains a smooth surface from afar (Figure 1Ag) but exhibits an irregular nanofibril structure upon closer inspection (Figure 1Bg), which is a typical for regenerated silk fibroin [8,9,10,11,12,17,18]. Blended films with different ratios are compared in sets in Figure 1A (100-μm scale bar) and Figure 1B (200-nm scale bar). When SF was mixed with PLLA at a ratio of 1:5 (SP-1/5), a dense network structure with pores was found (Figure 1Ab,Bb). Any raised spherulites of PLLA were not obvious in the morphology at this resolution. When the content of silk protein increases further (SP-3/5, SP-5/5), the morphology shows a regular convex–concave topology with pores becoming gradually smaller (Figure 1Ac,Ad). High magnification shows the compactness of the film as the SF content increases (Figure 1Bc,Bd). At even higher contents of SF (SP-5/3, SP-5/1), the surface morphology is uniform and smooth (Figure 1Ae,Af), with hardly any porosity (Figure 1Be,Bf). Instead, nanofibrous structures were well-embedded in the blended system at these ratios [17]. SEM images illustrated the silk fibroin blends with poly(L-lactic acid) homogeneously at the macroscopic level. In addition, the interlacing, bridged morphology between the two polymer chains can prevent crack propagation and improve the thermal stability and degradability of the materials [1,2,3,12,19].

### 2.2. Spectroscopy Analysis

**Figure 2:**
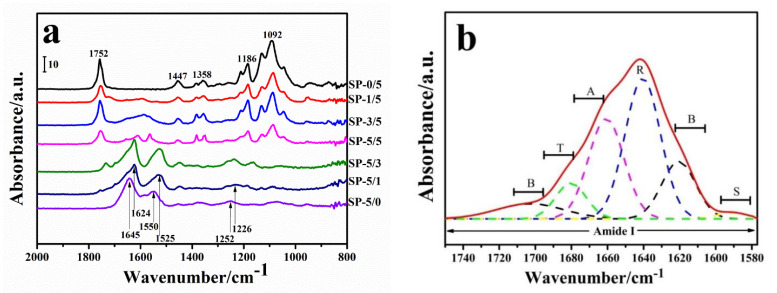
(**a**) Fourier-transform infrared (FTIR) absorbance spectra of the SF/PLLA blended films with different mass ratios (SF:PLLA = 0:5, 1:5, 3:5, 5:5, 5:3, 5:1, and 5:0) for untreated sample spectra in the 800~1900 cm^−1^ region; (**b**) A curve fitting example of the amide I spectra (sample SP5/1). The fitted peaks are shown by dashed lines and assigned as side chains (S), *β*-sheets (B), random coils (R), *α*-helix (A), and turns (T).

A FTIR analysis was used to reveal the secondary structure of proteins within the films. Figure 2 shows the FTIR spectra of SF/PLLA blended polymers with different mass ratios from 800 cm^−1^ to 2000 cm^−1^. For the raw PLLA sample (Figure 2a, SP-0/5), the absorption peak at 1752 cm^−1^ is the characteristic absorption band of C=O on the molecular chain [12,13]. The peaks at 1447 cm^−1^ and 1358 cm^−1^ are from the deformation and absorption vibration of C-H, respectively [1,2,14,17]. Both the 1186 cm^−1^ and the 1092 cm^−1^ peaks belong to C-O stretching vibrations [1,2,14,17]. For the pure SF sample (Figure 2a, SP-5/0), the main infrared spectral regions within 1700~1600 cm^−1^ and 1600~1500 cm^−1^ are assigned to the peptide backbone of the amide I and amide II absorptions, respectively [1,2,8,12,20]. The amide I region directly reveals the secondary structure of the protein backbone, which comes from C=O stretching vibrations [1,2,8,12,20]. The region 1600~1640 cm^−1^ is related to the protein *β*-sheet, which increases during silk crystallization. The region of 1640 cm^−1^~1660 cm^−1^ is dominated by vibrations from *α*-helices and random coils. The other parts of the spectra region (1660–1690 cm^−1^) are mainly from *β*-turn structures, with some small bands from other structures, including a possible *β*-sheet peak at 1690~1705 cm^−1^ [1,2,8,12,20]. The amide II bands are mainly from the out-of-phase combination of C-N stretching and N-H in-plane bending vibrations in the protein backbone [1,2,8]. The change of the microenvironment and the tertiary conformation of the proteins can be analyzed in the amide II region, which is often associated with the mixing of the vibrational modes of protein side-chain groups [1,21]. Some studies [1,17,22,23] reported that Ca^2+^ ions from the calcium chloride solvent could exchange with the hydrogen at the phenolic hydroxyl group of the tyrosine residue in the silkworm silk protein. This destroys the covalent bonds between the protein chains, which makes interactions between silk fibroin and polylactic acid occur more easily [1,17]. In this study, the absorption band peaks of the pure SF film (Figure 2a, SP-0/5) appeared at 1645 cm^−1^ (amide I) and 1541 cm^−1^ (amide II), which designate a random coil-dominated silk I structure [1,17,22,24]. By comparing the normalized FTIR curves of SP/PLLA blends (Figure 2a) with an increasing PLLA content, it was found that the absorption peak intensity at 1752 cm^−1^, 1186 cm^−1^, and 1092 cm^−1^ belonging to PLLA gradually increased, while the intensity of the peaks at 1645 cm^−1^ and 1541 cm^−1^ belonging to the silk fibroin gradually decreased [22]. Meanwhile, after blending SF with PLLA using the stirring and reflux condensation (SRC) method, many absorption peaks from individual components were shifted or changed. Specifically, the peak at 1252 cm^−1^ shifted to 1226 cm^−1^ when SF mixed with PLLA at a 1:5 ratio. The peaks at 1645 cm^−1^ and 1550 cm^−1^ in pure silk (SP-5/0) gradually shifted to 1624 cm^−1^ and 1525 cm^−1^ in sample SP-5/1, respectively, which indicates a silk I structure converting to a silk II structure. These findings indicated that new conformations appeared. Specifically, *β*-sheet crystals dominated the silk structure (at 1624 cm^−1^ and 1525 cm^−1^) in the SP-5/1 and SP-5/2 blended samples. This implied that the molecular interactions between SF and PLLA could lead to gradual conformation changes in the protein after blending [3,7,11,17].

In addition, the S-orbital of the electron acceptor and the P-orbital of the electron donor can effectively overlap through intermolecular actions between the two components. This causes a change in the original force constant of the chemical bonds and causes the position of the spectrum absorption peak to move [25,26,27,28,29]. In the case of SF/PLLA films, hydrogen bonding forms between C=O on the PLLA molecular chains and N-H on the SF chains. Earlier studies have shown how this causes the charge from the C=O double bond to delocalize to other atoms, reducing the double polarity and their absorption frequency [26,27,28,29], effectively shifting the characteristic peak of C=O to a lower wave direction [17,22,30,31,32]. The FTIR results in this study reflect this phenomenon. Other studies have also shown the occurrence of hydrogen bond interactions between SF and PLA, including in an electrospun composite [7]. Zhu et al. [17] further pointed out that the hydroxyls of amino acids such as *Ser*, *Glu*, and *Asp* on the SF chains could have strong interactions with the carbonyls of the PLA chains in composites of SF/PLA at the 98:2 to the 90:10 mixing ratios. This promoted the formation of intermolecular hydrogen bonds between the two molecules. Therefore, our results illustrated the strong hydrogen bond interactions between SF and PLLA in the polymer chains of their composites.

To further investigate the changes in different structures during the interaction between silk fibroin and PLLA in the blended films, a Gaussian fitting on the infrared spectrum of silk fibroin in the amide I region from 1750 cm^−1^ to 1580 cm^−1^ was performed. Figure 2a shows the FTIR spectra in the amide I and II regions of all the samples, while Figure 2b shows the amide I spectra of the SP-5/1 sample with fitted vibrational bands (red dotted line). The peak positions and their related secondary structures for silk proteins can be assigned from references [22,23] as *β*-sheets (B), random coil (R), alpha helix (A), turns (T), and side chains (S). Table 1 summarizes the percentage of different secondary structures for each untreated and methanol-treated SF-PLLA sample. For untreated pure silk fibroin films (SP-5/0), the *β*-sheet content was 23.29%, and the random coil and *α*-helix content was about 66.80%, which indicated that more random coiled structures were contained in the pure silk fibroin film [17,18,20]. When the PLLA content increased, the *β*-sheet content from silk fibroin decreased from 23.29% to 12.23%, while the content of random coils and *α*-helices increased from 66.80% to 73.89%, and turns increased from 8.55% to 12.56%. Meanwhile, the methanol treatment increased the *β*-sheet crystal content to 58.98% in the pure silk film (SP5/0). While composites showed lower *β*-sheet contents (12.23% in untreated SP1/5), the methanol treatment still increased their beta sheet crystallinity to 33.50 (Table 1). The amorphous phase decreased from 41.02% in pure silk fibroin (SP5/0) to 11.08% for the treated sample SP1/5. Xue et al. [22] believed that the *β*-sheet in silk proteins was important to promote the stability of protein molecules and the formation of insoluble structures. He et al. [33] found the *β*-sheet content decreased once the PLLA percentages encompassed more than 10% in an *Antheraea pernyi* Tussah silk fibroin/PLLA nanofiber composite. However, Taddei and coworkers in their earlier work [7] found no significant change in the *β*-content of SF after electrospinning with each other at 1:1 mix ratio. General, multilayer *β*-sheet crystals of silk fibroin fibers can be transformed to single-layer intramolecular *β*-sheets or random coils during dissolution in an acid system [23]. Once force and heat are applied to the protein solution, the disordered structure of SF can be transformed to crystals or order structures in return [12,23]. Similarly, in the study by Taddei et al. [7], the effects of both electrical force and solvent detachment force on the blends of silk fibroin and PLA during the spinning process caused changes in their structures. Thus, different post-treatments may affect the final conformation and structure of the composites on different levels. In this study, the content of *β*-sheet in the silk fibroin decreased with an increase in PLLA. Based on these references, this implies that the hydrogen bonds in the *β*-sheet crystals of silk fibroin molecules were partially broken due to the addition of PLLA, while a new hydrogen bond was formed with N-H on silk fibroin [1,7,17,20,30,32,33,34]. Simultaneously, the hydrophobic groups in silk fibroin could also generate electrostatic and hydrophobic interactions with polylactic acid segments [20,21].

**Figure 3:**
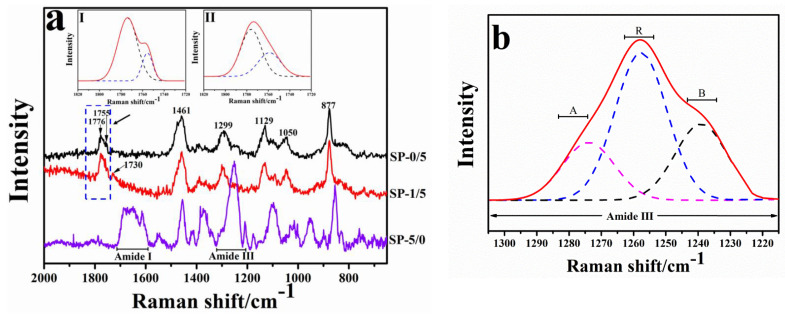
(**a**) Raman spectra of SF/PLLA blended films (SP-0/5, SP-1/5, and SP-5/0) with 0:5, 1:5, and 5:0 mass ratios of SF to PLLA. The inserted image (I) and image (II) showed enlarged Raman spectra of pure PLLA (SP-0/5) and the blending sample (SP-1/5) at 1720~1820cm^−1^, respectively, where the curve fitting peaks are shown at 1755 cm^−1^ and 1776 cm^−1^, respectively; (**b**) A curve fitting example of the amide III spectra (sample SP-1/5). The fitted peaks are shown by dashed lines and assigned as *α*-helix (A), *β*-sheets, (B) and random coils (R).

Furthermore, Raman spectroscopy was used to study the scattering phenomena during molecular interactions to supplement the infrared spectroscopy. For pure silk fibroin, the Raman spectrum is complex and diverse due to the different types of vibration models caused by peptide bonds (-CONH-)C-C and C-N backbones such as amide A; amide B; and amide bands I, II, III, IV, V, VI, and VII [22,23]. Among them, amide I and amide III are very significant bands owing to their high sensitivity for changes in protein conformation. The Raman spectrum characteristics of sample SP-5/0, blended sample SP-1/5, and sample SP-0/5 were compared in the 750~2000 cm^−1^ region (Figure 3). For pure silk fibroin (SP-5/0), the regions of 1229~1305 cm^−1^ and 1597~1680 cm^−1^ belong to the amide III and amide I regions, respectively. Specifically, 1645~1660 cm^−1^ in the amide I and 1265~1300 cm^−1^ in the amide III regions are assigned to *α*-helix, 1665~1680 cm^−1^ in amide I, 1230~1240 cm^−1^ in the amide III region are assigned to *β*-sheet, and 1660~1670 cm^−1^ in the amide I region and 1240~1260 cm^−1^ in the amide III region are assigned to random coil [24,25]. For raw PLLA (SP-0/5), the Raman spectrum shows a prominent absorption peak at 1776 cm^−1^ corresponding to C=O stretching vibrations on the PLA molecular chain. The absorption peaks at 1461 cm^−1^ and 1129 cm^−1^ correspond to the antisymmetric deformation vibration of -CH_3_. The absorption peaks at 1299 cm^−1^ and 1050 cm^−1^ belong to the bending vibration of -CH and the C-CH_3_ stretching vibration, individually, and the absorption peak at 877 cm^−1^ is attributed to the C-COO stretching vibration [17,26,28,30,31,32]. The Raman spectra characteristic peaks of both SF and PLLA are all shown in the blended film sample SP-1/5. Huang et al. [34] studied composites of polylactic acid (PLA) with montmorillonite and found that an absorption peak of C=O on the polylactic acid molecular chain at 1776 cm^−1^ would split into two peaks located at 1748 cm^−1^ and 1755 cm^−1^ when the crystallization temperature was higher than 140 ℃. They believed that intermolecular hydrogen bonding occurred between C=O on PLA and -CH_3_ on montmorillonite. Bruckmoser et al. [35] observed the crystallinity of the PLA fibers during melting and spinning by using the Raman spectra technique. They found that the spectra intensity of PLA decreased at 1755 cm^−1^ but increased at 1776 cm^−1^ when the stretching temperature and the stretching ratio increased. In this research, Figure 3a shows the enlarged regions of pure PLLA sample SP-0/5 and blended sample SP-1/5 in the 1720 cm^−1^–1820 cm^−1^ regions in inserted images I and II, respectively. Specifically, an obvious peak at 1775 cm^−1^ with the shoulder at 1755 cm^−1^ was observed on the spectrum curve of the pure PLLA (SP-0/5, image I inserted in Figure 3a), while on the blended sample curve (SP-1/5, image II inserted in Figure 3a), the little peak at 1755 cm^−1^ disappeared, and only one peak at 1775cm^−1^ was shown. Additionally, a new absorption peak at 1730 cm^−1^ was shown in the blended sample SP-1/5 after adding silk fibroin (Figure 3a). Furthermore, the spectrum intensity dropped from 80.60% to 64.25% at 1776 cm^−1^. These results may be attributed to the hydrogen bond formed between N-H and C=O from the proteins. This changes the bond length of C=O, lowering the vibration frequency to lower the wavenumber (Figure 3a) and the wave strength at 1776 cm^−1^. Moreover, by fitting a Raman spectrum curve at the amide III region of sample SP-1/5, the random coil, *β*-sheet, and *α*-helix contents in the amide III segment were calculated to be approximately 43.82%, 34.01%, and 22.17%, respectively. This indicates that the amorphous structure of silk fibroin dominated in the composite. All these findings are consistent with that of the FTIR spectrum discussion above. In summary, all results from the FTIR and Raman spectrum studies proved that SF and PLA have strong molecular interactions, which lead to new conformations after blending [1,13,33].

### 2.3. Phase Analysis

During the fabrication process, PLLA solutions were stirred under a magnetic field at a certain temperature [36,37,38,39]. Therefore, the magnetic force and temperature should play important roles in controlling the microstructure of the blended samples. The conditions of high force and high temperature can promote the extension of the molecular network and then crystallization in the polymer, since polymer chains in the amorphous phase are highly disordered. Some researchers [40,41,42,43] believed that a rigid amorphous fraction (RAF) exists in the amorphous phase with a degree of orientation in the polymer, where the RAF could not gain mobility like a regular mobile amorphous fraction (MAF) during the glass transition. RAF could not contribute to the heat fusion of crystal melting but could affect the glass transition and the stability of the polymer molecular chains. Many studies [40,41,42] reported that a kind of mesophase, just like the RAF, existed in some polymer block domains, including PLA, after they were melted while quenched or under an electrostatic field. Since X-ray powder diffraction (XRD) and differential scanning calorimetry (DSC) are useful techniques to study the crystallinity and phase structures of polymers, these two methods were adopted in the current work to investigate the phase structures of PLLA in the SF/PLLA composites.

**Figure 4:**
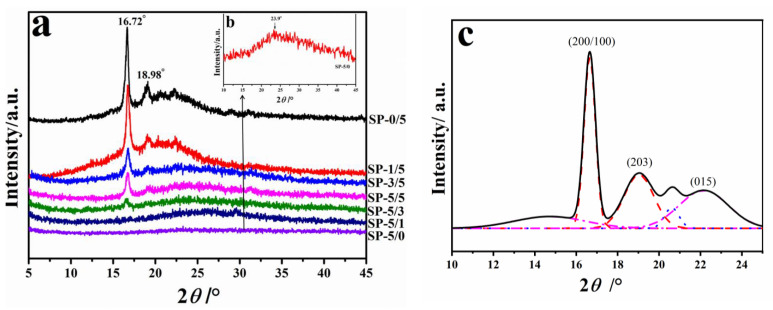
(**a**) x-ray diffraction (XRD) spectra of the SF/PLLA composite films (SP-0/5, SP-1/5, SP-3/5, SP-5/5, SP-5/3, SP-5/1, and SP-5/0) with different mass ratios (SF:PLLA = 0:5, 1:5, 3:5, 5:5, 5:3, 5:1, and 5:0); (**b**) The inserted graph shows the enlarged spectra of sample SP-5/0 and (**c**) deconvolution of the XRD peaks of the sample film SP-1/5. The solid curve is the best fit line (black). The fitted individual Gaussian peaks can be assigned to the crystal phase (red, dashed line), the mesophase (blue dotted line), and the amorphous phase (pink, dotted and short dashed line) [40,42,45,47].

Figure 4 shows the XRD spectrum of pure SF (SP-0/5); pure PLLA (SP-5/0); and their composite films (SP-1/5, SP-3/5, SP-5/5, SP-5/3, and SP-5/1). The pure PLLA sample (SP-0/5) has two sharp peaks overlayed on a broad and amorphous dispersion peak with the diffraction peak at ~16.7° (200/100) stronger than that at ~18.9° (203); both peaks are the characteristic peaks of the *α*-crystal form of PLA [34,35,36]. The inserted graph in Figure 4b shows the enlarged XRD spectra of the pure SF sample (SP-5/0) in the 10°~45° region. Xue et al. [23] investigated the five types of domestic and wild silkworm fibers and their regenerated films. They found that Mori silk fiber showed a peak at 20.4° in the wide-angle X-ray scattering curve, which was dominated by intermolecular *β*-sheet crystal structures. Its regenerated film, meanwhile, showed a broad peak centered at 24.3°, indicating intramolecular *β*-sheets and random coil structures typically associated with a silk I structure. The XRD measurement of our SF films showed a diffuse X-ray peak centered at 23.9° (Figure 4b), implying the presence of intramolecular *β*-sheets and large random coil structures. As the proportion of silk fibroin in the composite materials gradually increased, the intensity of the corresponding pure PLLA diffraction peaks at 16.7° and 18.9° gradually weakened, indicating that the crystallinity of the PLLA material declined gradually [44,45,46,47]. To better understand the crystal structure change of PLLA in SF-based blended polymers, a peak fitting calculation on the measured XRD curve was performed (Table 2). These calculated phases included the crystalline phase (*X*c), mobile amorphous phase (*X*_MAP_), and rigid amorphous phase (*X*_RAP_), which can all coexist in blended polymers [40,42]. The subscript -XRD was added to indicate that the data came from XRD measurements and calculations. Among them, the content of the mobile amorphous phase (*X*_MAP-XRD_) in the pure PLLA samples was up to 0.62, while the crystalline phase (*X*_C-XRD_) and rigid amorphous phase (*X*_RAP-XRD_) were 0.34 and 0.04, respectively. With the addition of silk fibroin into the composite, the content of the crystalline phase (*X*_C-XRD_) of PLLA gradually decreased, while, interestingly, the contents of the rigid amorphous phase (*X*_RAP-XRD_) gradually increased. For example, as the SF content ratio increased from 16.7% to 83.3%, the crystallinity decreased from 0.30 to 0.12, while the mobile amorphous phase increased from 0.64 to 0.77 and the RAF content increased from 0.04 to 0.11.

**Figure 5:**
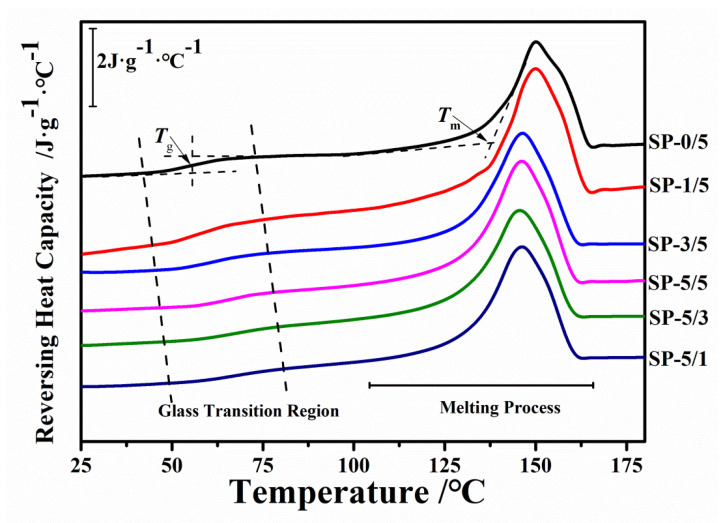
StepScan differential scanning calorimetry (SSDSC) reversible specific heat capacity curves of different composite films (SP-0/5, SP-1/5, SP-3/5, SP-5/5, SP-5/3, SP-5/1, and SP-5/0); the exothermic direction is downward.

Figure 5 shows the reversible specific heat capacity curves of the SP/PLLA composites obtained from StepScan differential scanning calorimetry (SSDSC). During the cooling process, the specific heat of the semicrystalline polymer materials changes abruptly when they change from the flowing liquid state to the original solid state, which is also seen in reverse [41,42,43]. During the initial region (25~100 °C) of heating, the specific heat has obvious discontinuous mutations, which show the glass transition region (with the glass transition temperature *T*_g_). In composite films, the glass transition temperature (*T*_g_) rises with the increase of the silk fibroin content, while the melting temperature and enthalpy of the composite is reduced (Table 2). This result implies that, with an increase in silk content, the amorphous phase structure in the silk fibroin composite tends to be more stable, and its potential barrier becomes larger, while the potential energy of the crystalized portions is lower and makes the material easier to melt [41]. Regardless, a single glass transition observed from each blend ratio indicates that the SF and PLLA blends are thermodynamically miscible at all ratios. In order to further verify the phase structure composition calculated by XRD, the specific heat increment and enthalpy were obtained from the DSC curve. The crystallinity (*X*_c-DSC_), mobile amorphous phase (*X*_MAP-DSC_), and rigid amorphous phase (*X*_RAP-DSC_) were also calculated and summarized in Table 2, according to the DSC methods described in the literature [40,42]. The crystallinity of these composite materials decreased with an increase of the silk fibroin ratio. Among them, the content of the amorphous phase in the PLLA samples compromised up to 0.64. The crystalline phase content of PLLA in the composites gradually decreased, and the rigid amorphous phase gradually increased with the addition of silk fibroin. For instance, compared with the pure PLLA (SP-0/5) sample, the crystallinity (*X*_c_) of the SP-5/1 composite polymer was lowered from 0.33 to 0.12, while the rigid amorphous phase increased from 0.04 to 0.11. This trend is consistent with the results from the above XRD analysis, indicating that the crystallinity in the composite material decreased while the amorphous content increased after silk fibroin was blended with PLLA. It is well-known that the melting point of a polymer material is related to its crystal structure and content. Similarly, the glass transition temperature is associated with the amorphous phase structure and motion within the polymer. Therefore, in this work, the reduction of the melting temperatures of the blended materials is attributed to the molecular rearrangement caused by interactions between silk fibroin and PLLA, as well as the reduction of the crystallinity of PLLA. Meanwhile, the glass transition region shifted towards higher temperatures due to an increase of the rigid amorphous phase content. In general, the DSC results correlate well with the XRD data to verify and supplement the conclusions from the FTIR and Raman spectrum discussions above. Together, all results indicated that clear interactions existed between silk fibroin and poly (L-lactic acid) in the blended films.

### 2.4. Thermal Stability

**Figure 6:**
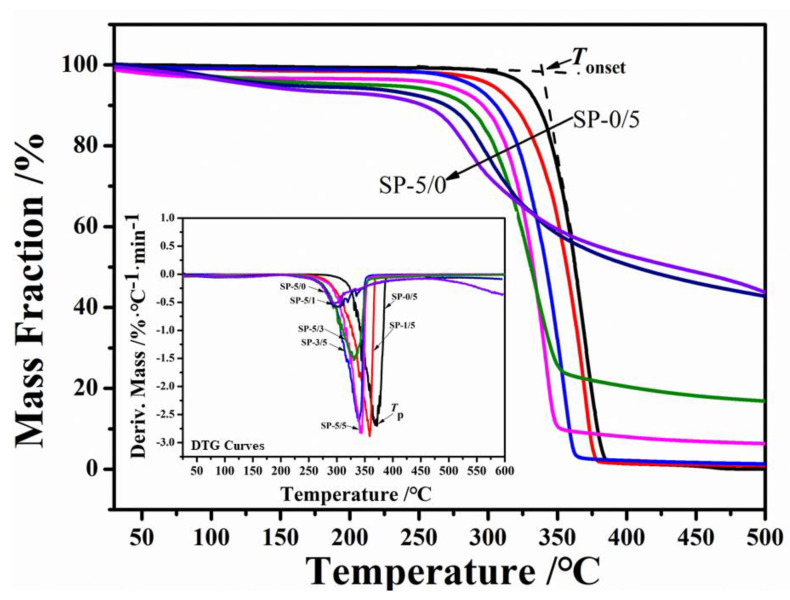
Thermogravimetric curves of SF/PLLA composite films heated from room temperature to 500 °C at 10 °C min^−1^. The insert graph shows the first derivative of the mass percentage curve, which shows the thermal decomposition rates of the different composite polymers (SP-0/5, SP-1/5, SP-3/5, SP-5/5, SP-5/3, SP-5/1, and SP-5/0).

The thermogravimetric (TG) analysis is one of the most important methods to study the thermal stability, decomposition, and structural composition of materials [48]. Figure 6 shows TG curves of the SF and PLA composite materials. During the initial heating from room temperature to 200 °C, all samples had a mass loss due to the evaporation of the bound solvent molecules or water. The water content (20~200 °C) increased significantly when silk was present, from 0.42% to 6.99%, which was ascribed to the better hydrophilicity and hygroscopicity of silk fibroin. On the other hand, the hygroscopicity of silk fibroin gradually decreased with the gradual addition of PLLA. During this initial heating phase, the pure PLLA film had almost no mass loss (0.42%) before it began to fully decompose. With the addition of silk fibroin, the mass loss increased to 1.73 wt % for SP-1/5, 4.48 wt % for SP-3/5, 4.80 wt % for SP-5/5, 5.52 wt % for SP-5/3, 6.07 wt % for SP-5/1, and 6.99 wt % for the pure SF (SP-5/0) before 200 °C (Table 3). After 200 °C, the TG curve displayed a stable and smooth process, with little loss of mass. Above 250 °C, all the samples showed one strong thermal degradation stage in the region of 250 °C–400 °C, which, in combination with the DSC results, indicated thermodynamic compatibility between silk fibroin and PLLA in the composites. With the addition of silk fibroin, the initial decomposition temperature (*T*_onset_) on the TG curves of the composite films tended to be lower temperatures, from 346.27 °C in pure PLLA to 272.76 °C in pure SF. At 450 °C, the residual amount of the sample decreased as the PLLA content increased. For example, the SP-5/0 film had about 48.77% remaining mass percent at 450 °C, the SP-5/5 sample about 7.15% remaining, and the SP-0/5 sample about 0.58% remaining. The degradation peak temperature (*T*_p_) shown in the derivative thermogravimetric (DTG) curve showed the maximum thermal decomposition rate of the samples. Clearly, SP-0/5 had the highest thermal decomposition rate temperature (371.34 °C) among all the samples. As higher amounts of silk were introduced into the composites, and for pure silk, the temperature decreased from 359.15 °C for SP-1/5 to 295.45 °C for SP-5/0. Additionally, sample SP-1/5 possessed the highest decomposition rate (2.89 wt %·°C^−1^) among all the blended films. The rate then gradually decreased with the increase of silk fibroin content from 2.62 wt %·°C^−1^ for sample SP-3/5 to 0.57 wt %·°C^−1^ for sample SP-5/0. Pure SF had the lowest degradation rate. These results imply that the SF and PLLA components formed a homogeneous mixture on the microscopic level mixed in the film systems [49]. This was further supported by how the degradation peak temperatures of the blend samples were different from the pure SF or pure PLLA curves. Above 400 °C, all the samples degraded uniformly, and no peaks appeared in the first derivative curves.

Overall, the TG results showed that the bound solvent contents in the SF/PLLA blended films were less than the pure SF film, and their thermal stability varied with the ratios of SF to PLLA in the films. Furthermore, the TG curves also illustrated that the SF and PLLA blended together homogenously on the microscopic level in the composite systems, which showed different thermal properties from the individual thermal profiles of SP-5/0 and SP-0/5 [50,51].

### 2.5. Interaction Mechanism

**Figure 7:**
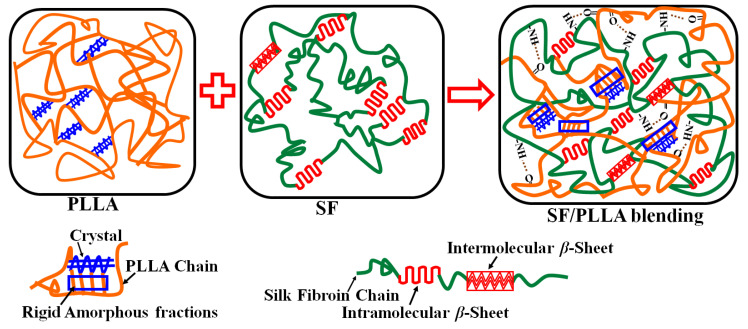
The schematic mechanism of interactions between SF and PLLA and their miscibility after blending.

In summary, polymer blending can synergistically combine the original components of the polymer system to control, achieve, or improve the various performances of polymer composite materials [8,9,10,52]. Figure 7 shows the schematic mechanisms of the interactions between SF and PLLA and their miscibility after blending. In this study, for the pure poly(lactic acid)-dichloromethane system, reflux condensation was used to fully mix poly(lactic acid) with dichloromethane due to the low boiling point of dichloromethane. Dichloromethane can form microdroplets in the cast membrane solution and gradually volatilize, through which a concentrated and viscous microenvironment is also produced [52,53]. Thus, a uniform network structure with tiny pores can be obtained after removing dichloromethane. Meanwhile, for pure fibroin in solution, CaCl_2_ molecules from the formic acid solvent can function as plasticizers in the system [23]. As a result, multilayer *β*-sheet crystals from silk fibroin fibers can be exfoliated into single-layer intramolecular *β*-sheets or random coils [23,54], forming a homogeneous fibroin solution. After the evaporation of the formic acid, the fibroin fibers assembled together into nanofibrils with a dominated structure of intramolecular *β*-sheets and random coils, which may indicate early-stage silk II or late-stage silk I structures [23]. Furthermore, the structure can also be transformed back into the intermolecular silk II with intermolecular *β*-sheet crystals if the polymer is annealed in water for a long time; treated with organic solvents such as methanol, ethanol, or mechanical stirring and pressing; or through thermal treatments [12,23,44,46,54]. Therefore, in this study, after SF and PLLA were blended with each other at a certain proportion and temperature using stirring and reflux condensation, the original exfoliated intramolecular *β*-sheets structure in the fibroin solution was able to partially transform to intermolecular *β*-sheet crystals due to mechanical forces and heat energy. When more silk was added to the blended system, the transformation percentage was increased; FTIR and Raman measurements verified this result. However, the secondary structure of silk fibroin in the blended system is still dominated by *α*-helix and random coil structures. On the other hand, when SF is mixed with PLLA, intramolecular hydrogen bonds in the silk fibroin structure can be ruptured, allowing the amide structure to be rearranged and the carbonyl in the PLLA molecule chains to stretch to form a special structure with three lone pair electrons. This allows the oxygen atom in the L-polylactic acid molecular chain to form an intermolecular hydrogen bond with the -NH in the silk fibroin peptide [27,30,31,32]. Thereby, the carbonyl group (C=O) in the polylactic acid molecular chain interacts with the amide group (R-NH) in silk fibroin to form a new hydrogen bond when they blend. In addition, the silk protein can facilitate various hydrogen bonds, polar–polar, and hydrophobic–hydrophobic interactions due to its copolymer structure, with the backbones comprised of alternating hydrophobic and hydrophilic domains at the nanoscale [35,37]. Therefore, the binding between hydrophobic PLLA chains and silk molecular domains can also be accomplished via hydrophobic–hydrophobic interactions. The combined FTIR, Raman, XRD, DSC, and TG analysis results proved these hypotheses. Moreover, silk fibroin with nanofibrils can also embed within the PLLA network structure when they blend, which results in micro-porosity in the morphology of the blended samples. The micropores can gradually disappear to create a smooth surface instead of a bumpy mesh as the content of silk fibroin increases. Additionally, the glass transition temperature increases owing to the mobile amorphous phase content increasing and the rigid amorphous phase existing. This morphology can be tuned by altering the ratio of SF to PLLA in the blended samples and through methanol treatment. After 20 min of methanol treatment, the percentage of *β*-sheets in the SF/PLLA sample decreased from 58.98% to 33.50% when the PLLA content was increased from 0% to 83.3%. Numerous studies [9,12,18,22,24,25,26,28,29] demonstrated that different microscopic molecular structures and macroscopic morphologies correlate to various properties of crystallizable polymer composite materials such as degradability, stability, and mechanical properties. In this work, the increase of PLLA crystallinity improved the thermal stability of the films and reduced the decomposition rate. The porous network structure also made the composite material more stable than pure silk fibroin, which possesses high hygroscopicity and is weak to degradation. Of course, the properties of the content-rich component can play a dominant role when two-phase materials interact [55]. Therefore, the differences in the material structure and performance can be used to prepare devices for various uses. For example, this technique can be used to make a bone repair scaffold with good thermal stability, or it can be used as a drug delivery vehicle with a sustained release of its drug load with a large amount of the amorphous phase. Based on this discussion, the SF/PLLA mixture is a thermodynamically miscible blend with uniform tunable thermal properties on a macroscopic scale.

## 3. Experimental Section

### 3.1. Preparation of SF/PLLA Films

The mulberry silkworm (Chinese *Bombyx mori*) was purchased from Dandong July Limited Trade Co. Ltd., Dandong, China. Poly(L-lactic acid) (PLLA) with glass transition temperature (*T*_g_): 60–65 °C, melting temperature (*T*_m_): 160 °C, and molecular weight (*M*_W_) of 80,000 Da was bought from Shenzhen Yisheng New Materials Co., Ltd., Shenzhen, China. Calcium chloride (CaCl_2_), formic acid (FA) and dichloromethane (DCM) were bought from XiLong Science Co., Ltd., Shantou, China. All reagents were of analytical grade. Regenerated silk and PLLA were weighted at the mass ratios of 0:5, 1:5, 3:5, 5:5, 5:3, 5:1, and 5:0 silk to PLLA. After being dissolved in 4.00 wt % CaCl_2_-FA solution and DMC solution, respectively, they formed 8.00 wt % silk fibroin (SF) solutions and 3.00 wt % PLLA solutions, individually. After blending with each other, the mixture solution was poured into a round-bottom flask and was stirred magnetically for 1.5 h while under reflex condensing at 45 °C. After blending using stirring and reflux condensation (SRC), the composites were cast in a polytetrafluoroethylene rectangular mold and vacuum-dried for 48 h at 35 °C to form the silk fibroin/Poly(L-lactic acid) (SF/PLLA) blended films. Finally, all films were soaked with deionized water to remove the solvent residues and then dried in a vacuum oven at 40 °C for 24 h. This investigation focused on the structure and thermal decomposition behavior of SF/PLLA films. The final SF/PLLA samples (SP-0/5, SP-1/5, SP-3/5, SP-5/5, SP-5/3, SP-5/1, and SP-5/0) were named according to the initial ratio of the silk fibroin and Poly(L-lactic acid) (0:5, 1:5, 3:5, 5:5, 5:3, 5:1, and 5:0, respectively) in the system solution, respectively.

### 3.2. Experimental Methods

Scanning electron microscopy (SEM) observation was performed using a JSM-7600F SEM from JEOL company (JEOL Ltd., Musashino, Akishima, Tokyo, Japan). The sample was placed in a sputter to be coated with gold for 10 s on each side, three times each, under 20-mA current, 10-kV working voltage, and 15-mm working distance. Fourier-transform infrared spectroscopy (FTIR) analysis was used on the NEXUS-670 FTIR spectrometer from Thermo Nicolet (Thermo Fisher Scientific Inc., Waltham, MA, USA).The sample was placed directly on the Ge crystal, and the OMNI sampler fixed button was rotated to press the sample tightly, and the single reflection ATR-OMNI was used. A spectral range with 4-cm^−1^ resolution, 32 scans, and 2000~900 cm^−1^ were used for infrared spectrum measurements. Laser Raman spectroscopy (Raman) analysis was completed on a HR800 French JY instrument (HORIBA Ltd., Kisshoin, Minamiku Kyoto, Japan). The He-Ne laser was used for an excitation light source. A scanning range of 735-nm excitation wavelength, 6-mW excitation power, 4-cm^−1^ resolution, 10-s integration time, and 650~2000 cm^−1^ was used for Raman spectrum measurements. X-ray diffraction (XRD) analysis was performed using the D/max 2500VL/PC type X-ray powder diffractometer (Rigaku Corporation, Matsubara-cho, Akishima-shi, Tokyo, Japan). The test conditions were CuK_α_ radiation, 40-kV tube pressure, 200-mA tube flow, and 5°~50°, with a 2θ diffraction angle range at 5°·min^−1^ scanning speed for testing. Differential scanning calorimetry (DSC) analysis (Diamond DSC, PerkinElmer, Waltham, MA, USA) was completed on both the standard DSC and StepScan DSC models from 25 °C to 220 °C at 5 °C·min^−1^ at isothermal increases of 1 °C·min^−1^ under nitrogen purge gas at 25 mL·min^−1^. Thermogravimetric (TG) measurements (Pyris 1 TGA, PerkinElmer, Waltham, MA, USA) was performed from 25 °C to 500 °C at a heating rate of 10 °C·min^−1^ under nitrogen purge gas at 50 mL·min^−1^.

## 4. Conclusions

SF/PLLA composite membranes with different mass ratios were prepared by reflux condensation and stirring blending. As the silk fibroin content in the composite membrane gradually increased, a regular circular convex–concave structure was shown on the porous junctions of the material surface. The more silk fibroin content present, the smoother the membrane surface was. The interaction between the molecules in the composite membrane mainly manifested through the formation of hydrogen bonds between amide groups on silk fibroin and C=O on the polylactic acid molecular chains. Simultaneously, hydrophobic and electrostatic interactions between the molecules of the blended materials also occurred. As the content of silk fibroin in the composite membrane increased, the glass transition temperature increased, while the melting temperature and enthalpy, as well as the crystallinity, gradually decreased. On the contrary, the addition of PLLA was able to help improve the thermal stability of the silk fibroin composites. This study provided us with a deep understanding of the microstructure transition and thermal stability of dual-crystallizable silk fibroin and poly(L-lactic) acid blended films, as well as their correlations at the molecular level. This information has practical significance for the design and fabrication of various biocompatible composite materials in the fields of tissue engineering, biomedicine, and biosensors.

## Figures and Tables

**Table 1:** Percentage of secondary structures in the silk fibroin/polylactic acid (SF/PLA) blended films.

Sample	*β*-Sheet (B) in Silk/ %	*a*-Helix & Random Coils in Silk/ %	Turns in Silk/ %	Side Chains in Silk/ %	Silk Amorphous in Sample/ %
SP-0/5	/	/	/	/	/
SP-1/5	12.23/33.50 ^a^	73.89	12.56	1.32	11.08
SP-3/5	13.48/42.80 ^a^	72.86	11.28	2.38	21.45
SP-5/5	15.54/44.32 ^a^	71.03	10.79	2.64	27.84
SP-5/3	17.03/45.03 ^a^	70.23	9.37	3.37	34.36
SP-5/1	18.24/48.26 ^a^	68.99	9.56	3.21	43.10
SP-5/0	23.29/58.98 ^a^	66.80	8.55	1.36	41.02

All calculated secondary structure fractions have the same unit (wt %) with ±2 wt % error. ^a^ *β*-sheet percentages after 20 min of methanol treatment.

**Table 2:** Thermodynamic parameters, crystallinities, mobile amorphous fractions, and rigid amorphous (mesophase) fractions in SF/PLA composite films.

Sample	SP-0/5	SP-1/5	SP-3/5	SP-5/5	SP-5/3	SP-5/1	SP-5/0
SF content (%)	0	16.7	37.5	50	62.5	83.3	100
*T*_g_ (°C)	55.81	58.68	62.31	65.02	72.03	74.33	154.32
*T*_m_ (°C)	150.29	150.01	148.93	147.11	145.57	144.98	/
Δ*H*_m_ (J·g^−1^)	30.23	25.37	23.56	18.68	13.95	10.23	/
Δ*C*_P_ (J·g^−1^·°C^−1^)	0.39	0.41	0.42	0.44	0.45	0.47	/
*X*_C-DSC_	0.33	0.27	0.24	0.19	0.15	0.11	/
*X*_MAP-DSC_	0.64	0.67	0.68	0.72	0.74	0.77	/
*X*_RAP-DSC_	0.03	0.06	0.08	0.09	0.11	0.12	/
*X*_C-XRD_	0.34	0.30	0.25	0.21	0.16	0.12	/
*X*_MAP-XRD_	0.62	0.64	0.68	0.70	0.73	0.77	/
*X*_RAP-XRD_	0.04	0.06	0.07	0.09	0.10	0.11	/

*T*_g_, *T*_m_, and Δ*H*_m_ refer to the glass transition temperature, melting temperature, and melting enthalpy, respectively. The heat capacity increment of 100% amorphous PLLA at *T*_g_ is 0.61 J·g^−1^·°C^−1^ [40,48]; the melting enthalpy of 100% crystalline PLLA is reported to be 93 J·g^−1^ in the previous literature [40,41,47,48]. *X*_C_, *X*_MAF_, and *X*_RAF_ are the three phase fractions that are crystallinity, the mobile amorphous fraction, and the rigid amorphous fraction. Subscripts –DSC and –XRD represent that the calculated results were obtained from the differential scanning calorimetry (DSC) and X-ray diffraction (XRD) curves, individually.

**Table 3:** Thermal decomposition parameters of the SF/PLLA films from the thermogravimetric (TG) analysis.

Sample	SP-0/5	SP-1/5	SP-3/5	SP-5/5	SP-5/3	SP-5/1	SP-5/0
SF content(%)	0	16.7	37.5	50	62.5	83.3	100
*T*_onset_ (°C)	346.2^7^	330.1^9^	319.2^5^	305.3^8^	292.1^7^	283.3^7^	272.7^6^
*T*_p_(°C)	371.3^4^	359.1^5^	338.5^0^	342.2^3^	330.9^1^	300.5^6^	295.4^5^
Δ*Y*_w_ (%)	0.4^2^	1.7^3^	4.4^8^	4.8^0^	5.5^2^	6.0^7^	6.9^9^
*Y_450_* (%)	0.5^8^	1.0^2^	1.8^9^	7.1^5^	18.1^0^	46.1^4^	48.7^7^
*v_P_* (wt %·°C^−1^)	2.7^1^	2.8^9^	2.8^4^	2.6^2^	1.5^4^	0.5^7^	0.5^2^

*T*_onset_, *T*_p_, and Δ*Y*_w_ are the initial decomposition temperature, the peak temperature of the main decomposition in the thermogravimetric derivative curve, and the water content in blended composites in the 20~200 °C region, respectively, which are derived from the TG measurements. *Y_450_* represents the residual amount of the sample at 450 °C. *v_P_* stands for the decomposition rate at the *T*_p_ temperature, which is the peak height in the derivative thermogravimetric (DTG) curve (with error bars less than 5%).
